# Design of Quad-Port Ultra-Wideband Multiple-Input-Multiple-Output Antenna with Wide Axial-Ratio Bandwidth

**DOI:** 10.3390/s20041174

**Published:** 2020-02-20

**Authors:** Pawan Kumar, Shabana Urooj, Areej Malibari

**Affiliations:** 1 Department of Electrical Engineering, School of Engineering, Gautam Buddha University, Greater Noida 201312, India; 2 Department of Electrical Engineering, College of Engineering, Princess Nourah Bint Abdulrahman University, Riyadh 84428, Saudi Arabia; 3 Department of Computer Science, Faculty of Computing and IT, King Abdulaziz University, Jeddah 80200, Saudi Arabia; aamalibari1@kau.edu.sa; 4 College of Engineering, Princess Nourah Bint Abdulrahman University, Riyadh 84428, Saudi Arabia

**Keywords:** circularly polarized, compact, connected ground, MIMO, planar, UWB

## Abstract

This article presents a compact, planar, quad-port ultra-wideband (UWB) multiple-input–multiple-output (MIMO) antenna with wide axial ratio bandwidth (ARBW). The proposed MIMO design consists of four identical square-shaped antenna elements, where each element is made up of a circular slotted ground plane and feed by a 50 Ω microstrip line. The circular polarization is achieved using a protruding hexagonal stub from the ground plane. The four elements of the MIMO antenna are placed orthogonally to each other to obtain high inter-element isolation. FR-4 dielectric substrate of size 45 × 45 × 1.6 mm^3^ is used for the antenna prototype, and a good agreement is noticed among the simulated and experimental results. The proposed MIMO antenna shows 3-dB ARBW of 52% (3.8–6.5 GHz) and impedance bandwidth (S_11_ ≤ −10 dB) of 144% (2.2–13.5 GHz).

## 1. Introduction

The increasing demand of planar antennas for wireless applications is due to the numerous advantages they offer, such as simple configuration, low-profile, compact size, low cost, and easy integration into portable devices [1,2]. They can be utilized for rapid communication applications, which involves a high transmission rate, for example, peer-to-peer data transfer and short-range wireless transmissions [3,4]. Recently, the attention of the researchers has been shifted to multiple-input–multiple-output (MIMO) antennas as they help in reducing multipath fading and achieving higher range, more reliability, and high data rate, without increasing transmitted power or bandwidth [5]. Several individual elements are combined together, with enough inter-element isolation, to obtain a high-performance MIMO/diversity antenna [6]. Due to space limitations in the wireless devices, there is a requirement for small-sized MIMO antenna configurations, which may result in a larger mutual coupling effect and subsequent degradation in the performance of the system [7]. Recently, researchers reported several MIMO antennas with compact size and high inter-element isolation [8,9,10,11,12]. A dual-port antenna with a common ground was presented in [8], where high isolation between the two antenna elements was obtained through the slotted ground plane. In [9], a compact antenna comprising of the complementary split-ring resonator (CSRR) implanted ground surface and two radiating elements was proposed for ultra-wideband (UWB). UWB antennas are commonly used for tracking objects and finding their accurate location. These antennas use short pulse methods to achieve high range resolution, as the performance of short pulse structures is superior in multipath environments. UWB antennas have proved their effectiveness in wireless sensor networks (WSNs) and body area networks (BANs), which need low transmission power [10,11]. Since UWB antennas use the frequency range from 3.1–10.6 GHz, some application bands that exist within this range may face interference issues. Thus, it is important to eliminate bands that cause interference. Numerous types of band elimination antennas have been reported to reject frequently used applications like WLAN, Wi-MAX, Bluetooth, etc. [12,13]. Additionally, spatial diversity schemes are used with UWB structures to improve the system’s performance. Antenna diversity is a recognized technique to improve the performance of UWB systems by encountering the multipath fading and co-channel interference [14,15]. Introducing more antenna elements at the transmitting/receiving terminals increases the link reliability of the transceiving system, thus making the receiver extra robust, efficient, and secure. The UWB and MIMO technology grouping supports high data rate transmission with the least multipath fading, accordingly, increasing the reliability and transmission capacity of the communication system. In [16], a dual-band MIMO antenna containing two upturned F-shaped radiators was presented for the WLAN application. A quad-element MIMO antenna composed of the split-ring resonator and inverted L-shaped radiating elements was proposed for wideband operation [17]. In [18], a quad-element MIMO antenna system comprising of concentric square-ring patches was explored, where isolation was attained by etching a pair of CSRRs on the ground surface.

Devices with circularly polarized (CP) MIMO antennas are in high demand and find applications in RFID, radar, satellite, Bluetooth, WLAN, and Wi-MAX systems [19]. A CP MIMO transceiver system does not suffer from the polarization mismatch problem amongst the transmitter antenna and the receiver antenna, therefore it possesses large spectral efficiency. The problem of mutual coupling can be solved more efficiently by using a MIMO configuration with polarization diversity. However, only a few MIMO antenna designs were suggested with circular polarization features. In [20], a dual-element MIMO antenna system using polarization diversity property was proposed, where a tunable metal strip loaded on the ground surface controlled the phase variation amongst the two modes. A dual-port antenna with Minkowski island curves, Koch curve fractals, and rectangular slotted ground plane laden with T-shape strip to obtain a higher level of inter-element separation was suggested [21]. In [22], a CP dual-port MIMO/diversity antenna was presented, where the ground surface was loaded with a cross branch structure to obtain polarization diversity. A two-element MIMO antenna, with one element at the upper surface and another element at the lower surface of the substrate, was proposed [23], where the top radiator emits CP waves and the bottom radiator emits linearly polarized (LP) waves. In [24], a compact CP two-element MIMO antenna was presented for off-body communications, where a stub extended from the ground surface alongside the feed line produces the vertical component, while the current alongside the width of the ground surface produces the horizontal components. A CP dual-port MIMO antenna was suggested for 1.8 to 2.6 GHz bandwidth in [25], where a line patch was embedded between the radiating patches to obtain high isolation. In [26], a compact dual polarized UWB MIMO antenna for access point applications was proposed, where a modified serpentine structure (MSS) decoupling element was implanted amongst the radiators to obtain high isolation. A wideband CP dielectric resonator-based MIMO antenna was suggested in [27], where isolation between the elements was enhanced by means of a parasitic patch and arranging the resonators diagonally. In [28], a compact three-port MIMO antenna comprising of one patch antenna and two dipole antennas was suggested, where the CP band was realized by means of truncated corners. An antenna composed of four rectangular-shaped radiating elements with truncated corners and cross-shaped slots loaded in their centre was designed [29], where a two-arm feeding mechanism was used to divide power between the radiators. A four-element CP MIMO antenna with truncated corners was proposed for triple-band operation [30], where one band emits LP waves and two bands emit CP waves. In [31], an antenna comprised of four orthogonally arranged triangular-shaped symmetrical radiating elements was presented, where a neutralization ring was used for reducing the inter-element coupling. An aperture coupled, multi-layered, cross-slot antenna design with circular polarization characteristics was proposed [32], where mutual coupling suppression was achieved using a double-layer transmission kind frequency selective surface (FSS) superstrate. However, the existence of complex elements in most of the above-reported antenna designs increase their overall size and complexity, and the integration of the proposed antennas into the monolithic microwave integrated circuits (MMICs) becomes difficult.

This communication presents a planar CP UWB MIMO antenna consisting of four identical radiators fed through 50 Ω microstrip lines. A wide circular-shaped slot is loaded on the ground surface of each element to obtain UWB, and a hexagonal-shaped stub protruding from the ground surface of the antenna element obtains circular polarization. The ground planes of the four antenna elements are linked to each other through a pair of metal strips, which results in achieving the same reference level and correct interpretation of all the signals. The presence of non-connected or separated ground planes may limit the application of antenna in many practical wireless applications as all the ground surfaces would not be at the same voltage level. Moreover, the decoupling methods based on the defected ground plane (reported in the literature) hold their validity only when the ground planes of the antenna radiators are not completely separated [33]. The antenna presented in this paper is of small size and possesses very wide 3-dB axial ratio bandwidth (ARBW) and (S_11_ ≤ −10 dB) impedance bandwidth. The antenna demonstrates high inter-element isolation and good diversity performance, making it a superior candidate for UWB MIMO systems and wireless personal area networks.

## 2. Antenna Design

### 2.1. Antenna Element Design

**Figure 1:**
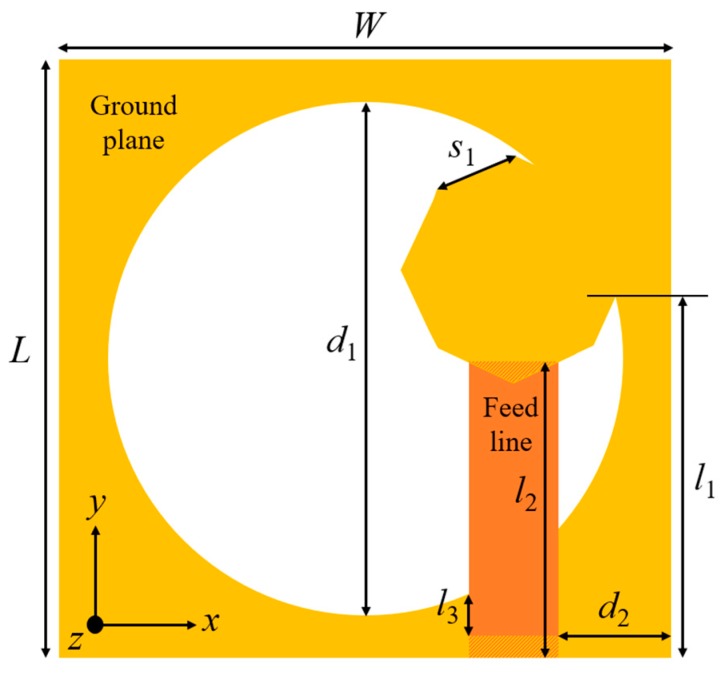
Diagrammatic layout of the slot antenna.

The diagrammatic layout of the slot antenna is displayed in Figure 1. The antenna contains a ground plane at the lower edge and a microstrip line at the upper edge of the dielectric substrate. A wide circular-shaped slot is introduced on the ground surface to obtain UWB. The circular polarization is obtained by a hexagonal-shaped stub bulged from the ground surface. The hexagonal stub establishes a 90° phase difference between the E-field components of the same magnitudes. The antenna is excited using a feed line of 50 Ω, which is positioned in the vicinity to the hexagonal stub, for broadening ARBW of the single element. Low-cost FR-4 substrate with 1.6 mm depth, loss tangent of 0.02, and relative permittivity of 4.4 is used for the antenna designing. The dimensions of the design elements are mentioned in Table 1. A 3-D electromagnetic simulation software (ANSYS HFSS^®^) is utilized for the simulation and designing of the antenna structures.

**Figure 2:**
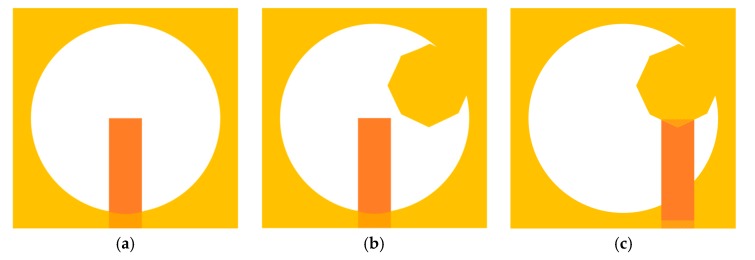
Evolution stages of the slot antenna: (**a**) Stage-1; (**b**) stage-2; (**c**) stage-3.

The sequence of the design steps (for a single element of the antenna) is demonstrated in Figure 2. The side edge of the square-shaped patch is evaluated as [34]

(1) $$f_{c}=\frac{c}{2\sqrt{\varepsilon_{r}}}\sqrt{{\left(\frac{m}{L}\right)}^{2}+{\left(\frac{n}{W}\right)}^{2}}$$

where *f_c_* denotes the centre frequency, *ε_r_* denotes the relative permittivity of the dielectric material, and *c* denotes the speed of light in vacuum. *L* and *W* denote the square-shaped patch length and width, respectively, and *m* and *n* denotes the dominant modes. Assuming the centre frequency of the square element as 7 GHz for the dominant mode (*m* = 1 and *n* = 0), the length of the antenna element (*λ*/2) is calculated as 20 mm.

The thickness of the feeding line is evaluated as [35]

(2) $$w_{f}=\frac{7.48\times h}{e^{\left(Z_{0}\frac{\sqrt{\varepsilon_{r}+1.41}}{87}\right)}}-1.25\times t$$

where *Z*_0_ denotes the input impedance, *h* denotes the dielectric substrate thickness, and *t* is the copper thickness. The width of the feed line is evaluated as 3 mm, using *h* = 1.6 mm, *Z*_0_ = 50 Ω, and *t* = 1 oz.

Firstly, a customary microstrip line-fed square-shaped antenna is designed for a resonating frequency of 7 GHz. In the next step, as displayed in Figure 2a (stage-1), a wide circular slot is etched from the square-shaped patch, which is fed by a centrally positioned microstrip feed line. Varying dimensions of the etched slot optimize the current path or the resonating frequency band for the presented antenna. Then, a hexagonal-shaped stub is bulged towards the centre of the etched slot, as illustrated in Figure 2b (stage-2). The circular polarization performance can be achieved by circular, rectangular, or rhombus-shaped stub. However, as compared to the mentioned geometries, the electrical length of the hexagonal stub is larger, therefore the surface current path is also larger, which leads to wider impedance and ARBWs. Figure 2c shows stage-3 of the proposed slot antenna, which involves shifting of the feed line towards protruded hexagonal stub. The location of the feed line at the centre of the wide slot (stage-2) results in the symmetrical distribution of EM energy throughout the slot. A significant improvement in impedance matching is noticed by changing the position of the feed line (in stage-3). The hexagonal stub in the ground surface and offset feed line position helps in achieving circular polarization characteristics in the antenna operating range. Furthermore, the coupling among the feed line and the ground surface is reduced by etching some part of the ground surface underneath the microstrip line feed.

**Figure 3:**
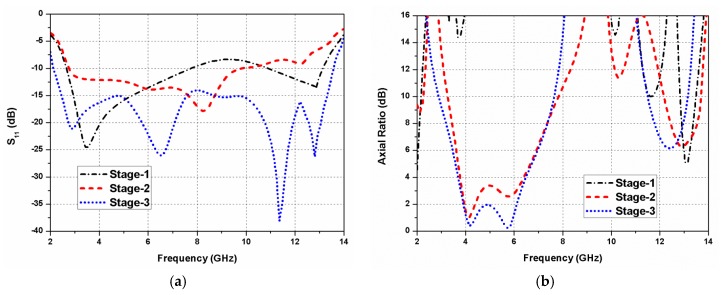
Simulated results of the evolution steps: (**a**) Reflection coefficients; (**b**) Axial ratio.

The comparison of reflection coefficients for different design steps (shown in Figure 2) is given in Figure 3a. The antenna designing starts with the square-shaped antenna, where the dominant mode resonates. Thereafter, a wide circular slot is embedded in the ground surface of the square-shaped element, which introduces dual mode resonances. The stage-1, consisting of a 50 Ω microstrip line and a wide circular slotted ground surface, possesses a wide impedance bandwidth. Furthermore, the protruded hexagonal-shaped stub introduces the third resonance. Finally, a wideband performance is obtained by the integration of these three resonances. The impedance matching is improved by changing the location of the feeding line, and by introducing a rectangular slot (in the ground plane) under the feed line.

The comparison of the axial ratio for different design stages of the slot antenna is shown in Figure 3b. Stage-1, having circular slotted ground plane structure, is seen to possess linear polarization characteristics. In stage-2, the introduction of a hexagonal stub increases the current path of the antenna; consequently, an improvement in the impedance bandwidth is noticed. Moreover, the hexagonal stub establishes a 90° phase difference between *E_xoy_* and *E_yoz_* components needed for achieving circular polarization characteristics. It is well known that E-field components of the same magnitudes and a quadrature phase variance gives rise to CP radiation [36,37]. In the proposed antenna element, the hexagonal stub introduces a 90° phase difference amongst the two (horizontal and vertical) electric field components. As a result, a subsequent reduction in the axial ratio is noticed, and it is seen to be 1 dB in the operating range of the antenna. A considerable improvement in the 3-dB ARBW is achieved by positioning the feed line under the hexagonal stub. Additionally, a part is carved out from the ground surface underneath the feed line, which decreases the coupling level between the ground plane and the feed line, hence improving the circular polarization characteristics and impedance bandwidth of the antenna element.

**Figure 4:**
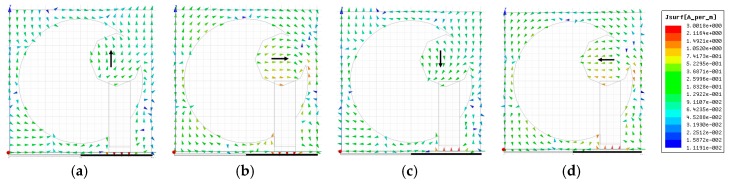
Current distribution at 5.75 GHz: (**a**) *ωt* = 0°; (**b**) *ωt* = 90°; (**c**) *ωt* = 180°; (**d**) *ωt* = 270°.

**Figure 5:**
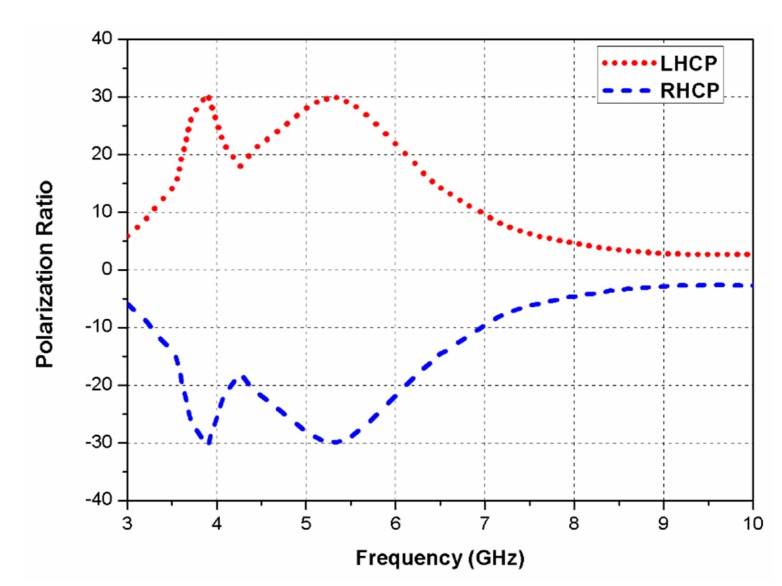
Polarization ratio of the proposed slot antenna.

The surface current distributions of the slot antenna (shown in Figure 1) at 5.75 GHz are illustrated in Figure 4. The field concentrated in the protruded hexagonal-shaped stub affects the circular polarization performance of the antenna. The current distribution around the hexagonal stub is checked at different time instants (*ωt* = 0°, 90°, 180°, and 270°), and a traveling wave rotating in the clockwise direction is noticed, which validates left-hand circularly polarized (LHCP) behaviour of the proposed antenna element. Furthermore, Figure 5 shows the polarization ratio of the proposed antenna element, which also verifies its LHCP operation. The curves in Figure 5 illustrates the purity of circular polarization and indicates that the magnitude of LHCP is remarkably greater than the RHCP. Unlike other CP antennas reported in the literature, the slot antenna presented is low-profile, compact-sized, and can be designed and fabricated easily as it does not include a loading of any active components or patch truncation. The proposed design shows an ARBW of 53% (3.7–6.4 GHz) and ultra-wide impedance bandwidth of 144% (2.2–13.6 GHz).

### 2.2. CP UWB MIMO Antenna Design

**Figure 6:**
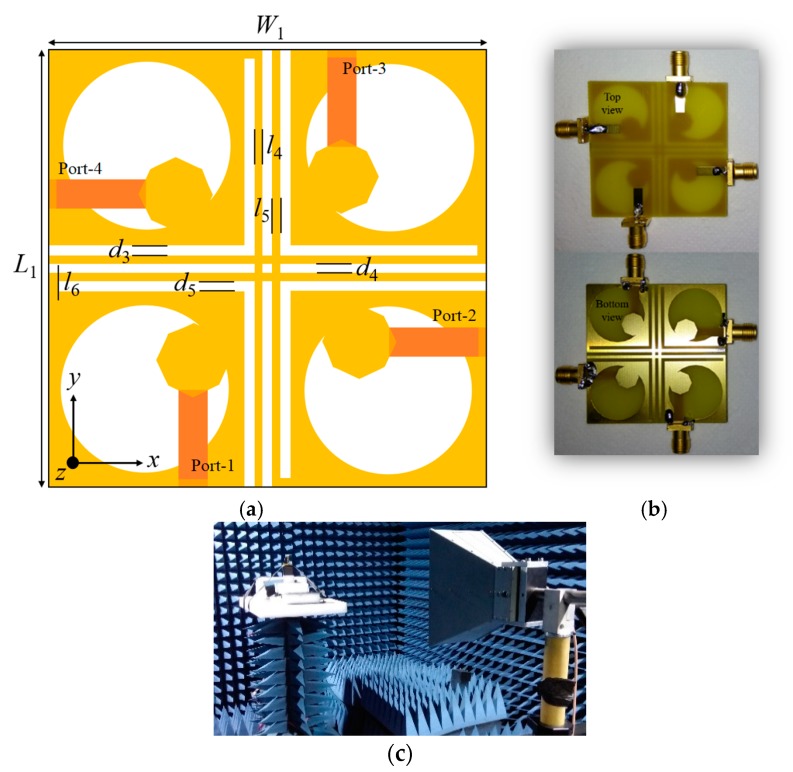
Proposed circularly polarized (CP) ultra-wideband (UWB) MIMO antenna: (**a**) Layout; (**b**) prototype; (**c**) measurement in anechoic chamber.

The MIMO design is illustrated in Figure 6a and the fabricated prototype (front view and rear view) is presented in Figure 6b. The proposed quad-port MIMO antenna is comprised of four antenna elements arranged orthogonally and are separated using metallic strips and space. The mutual coupling depends on the magnitude of the distance amongst the radiating elements. Changing the distance among four radiating elements does not considerably alter the resonating bandwidth of the MIMO antenna. However, the S*_ji_* (*i* ≠ *j*) values increase with less spacing between the elements, which indicates a stronger coupling among them. Furthermore, the ARBW is highly influenced by the spacing amongst the radiating elements. When the spacing is reduced, the axial ratio shifts towards a higher frequency and the percentage of ARBW decreases. A pair of metallic strips are introduced between the ground planes, which not only helps in connecting the ground planes with reduced inter-element coupling, but also helps in restoring ARBW. The total size of the presented UWB MIMO antenna is 45 × 45 × 1.6 mm^3^. The proposed UWB MIMO antenna prototype measurement set up inside an anechoic chamber is displayed in Figure 6c.

**Figure 7:**
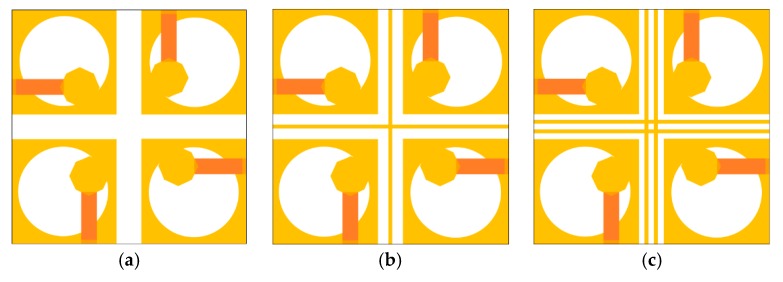

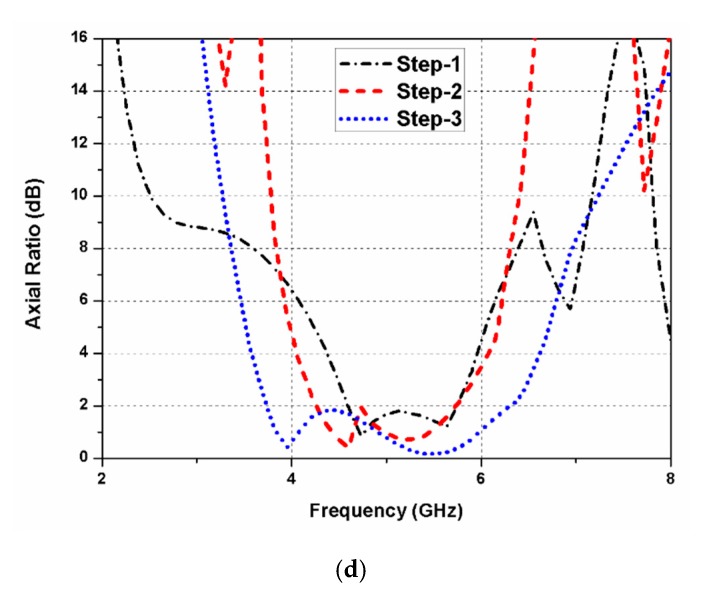
Proposed CP UWB MIMO antenna design steps: (**a**) Step 1; (**b**) step 2; **(c)** step 3; (**d**) axial ratio comparison.

The evolution and axial ratio comparison of the MIMO antenna design steps are displayed in Figure 7. In the proposed work, the main objective is to obtain a broad ARBW, when the ground planes of the four elements are linked together. However, it is very challenging to uphold ARBW of the antenna elements in the ground radiating multi-antenna arrangement. This is because the connecting strips are made up of metal, therefore they will also face coupling problems and leak current towards the nearby radiating elements.

Firstly, as displayed in Figure 7a, four square-shaped antenna elements are arranged orthogonally on the dielectric substrate of size *L*_1_ × *W*_1_ (step-1). In MIMO systems, the mutual coupling can be reduced by introducing space amongst the antenna elements. However, a large spacing among the radiating elements is not favoured, since it increases the physical dimensions of the MIMO antenna. Therefore, in the proposed geometry, a small gap is introduced among the antenna elements to uphold ARBW of the radiating elements. The inter-element isolation among antenna elements can be further improved through decoupling components. A plus-shaped metal strip is introduced in the centre of the MIMO geometry (step-2) to reduce interference among the neighbouring antenna elements, as illustrated in Figure 7b. The plus-shaped strip improves ARBW of the MIMO antenna (shown in Figure 7d) by reducing the inter-element coupling further. However, to restore ARBW of the antenna element completely, more isolation between the radiating elements is desired. Therefore, one more metal strip of similar dimensions (shown in the previous step) is introduced between the antenna elements, as shown in Figure 7c. With this additional strip, there are two coupling paths between the antenna elements. The first one is the original coupling path that happens between the antenna elements (step-2), while the other path created in step-3 is through the additional metal strip. The coupling current on the additional metal strip is approximately 180° out of phase with the original coupling current. The MIMO geometry shown in step-3 improves ARBW of the antenna element significantly. The spacing and location of the metal strips are adjusted in such a way that a compact MIMO geometry (with least inter-element coupling and broad ARBW) is obtained. The final proposed MIMO antenna geometry, with linked ground planes of the antenna elements, is shown in Figure 6a.

## 3. Results Discussion

**Figure 8:**
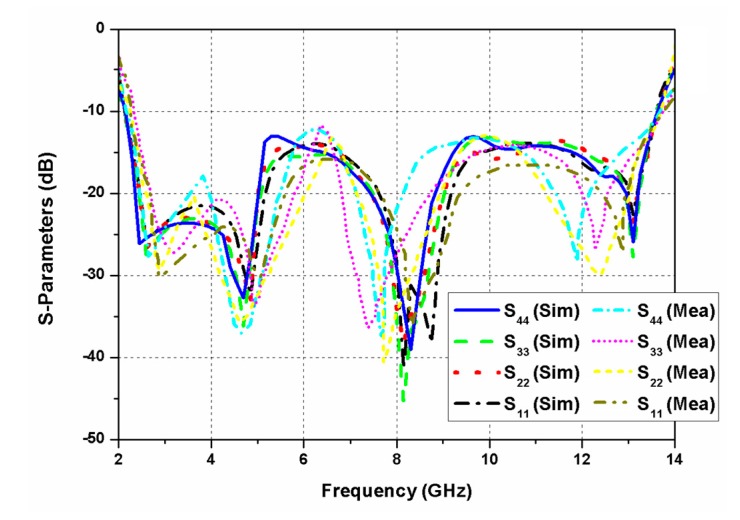
S-parameters (simulated and measured) of the proposed CP UWB MIMO antenna.

**Figure 9:**
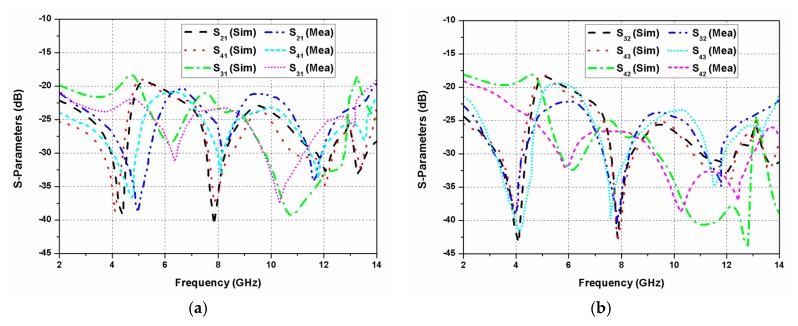
S-parameters (simulated and measured) of the proposed CP UWB MIMO antenna at (**a**) port-1; (**b**) other ports.

The fabrication of the antenna was performed using the photoetching process. Agilent N5230A PNA-L vector network analyser was utilised to measure various antenna parameters. The simulated and experimental values of the reflection coefficients are given in Figure 8. The proposed MIMO antenna worked in the band of 2.2 to 13.5 GHz (11.3 GHz), hence covering the entire UWB. The electrical performance of the antenna was measured using 50 Ω SMA connectors. During the measurement process at one port, the other antenna ports were matched with a 50 Ω load. The isolation between different antenna elements of the MIMO configuration is presented in Figure 9. The isolation value between different antenna ports was seen to be greater than 18 dB.

**Figure 10:**
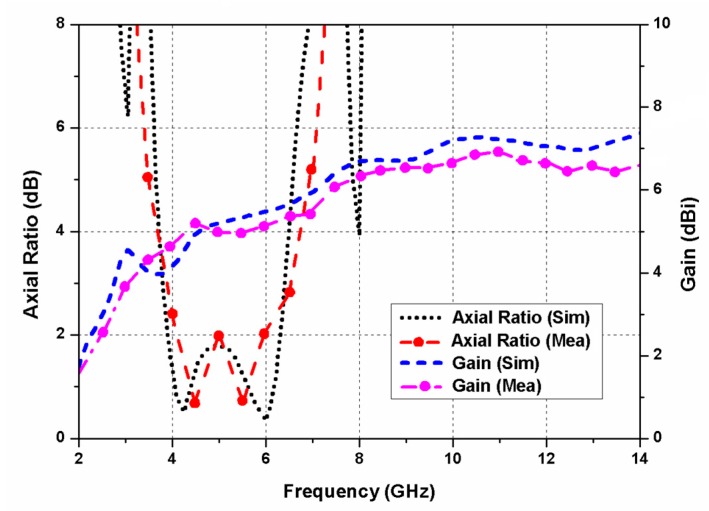
Axial ratio and gain (simulated and measured) of the designed CP UWB MIMO antenna.

Figure 10 illustrates the axial ratio response of the designed MIMO/diversity antenna, and superior quality of circular polarization (3.8–6.5 GHz) was seen in its resonating range. The introduction of spacing between the antenna elements restored the ARBW. The usage of decoupling metal strips between the ground planes increased the inter-element separation and also preserved the ARBW of the proposed antenna. The addition of decoupling structures and spacing may have increased the design complexity and overall antenna size. Therefore, to maintain a small size of the CP antenna, optimizations of the spacing and strip width were performed.

The simulated and measured gain results are displayed in Figure 10. The peak gain of the designed CP antenna was 6.8 dBi. The envelope correlation coefficient (ECC) for a quad-port diversity system (Figure 6, port-1 and port-2) was evaluated as [38]

(3) $$\rho_{e}\left(1,\;2,\;4\right)=\frac{{\left|S_{11}^{*}S_{12}+\;S_{21}^{*}S_{22}+S_{13}^{*}S_{32}+\;S_{14}^{*}S_{42}\right|}^{2}}{\left(1-{\left|S_{11}\right|}^{2}-{\left|S_{21}\right|}^{2}-{\left|S_{31}\right|}^{2}-{\left|S_{41}\right|}^{2}\right)\left(1-{\left|S_{12}\right|}^{2}-{\left|S_{22}\right|}^{2}-{\left|S_{32}\right|}^{2}-{\left|S_{42}\right|}^{2}\right)}$$

**Figure 11:**
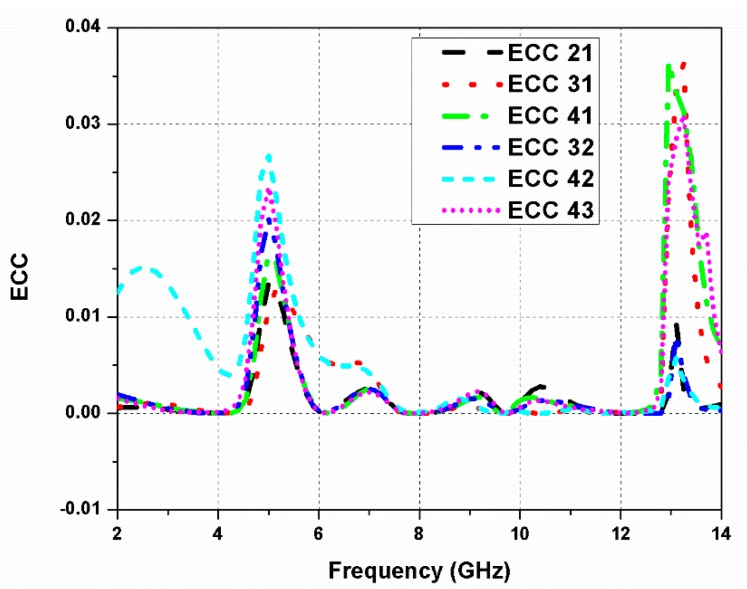
Envelope correlation coefficient (ECC) of the proposed quad-port UWB MIMO antenna.

Similarly, the values of *i* and *j* can be changed to calculate the ECC between other antenna ports. Figure 11 shows the antenna diversity performance, and ECC values were seen as less than 0.04 for the whole operating range of the designed MIMO antenna.

**Figure 12:**
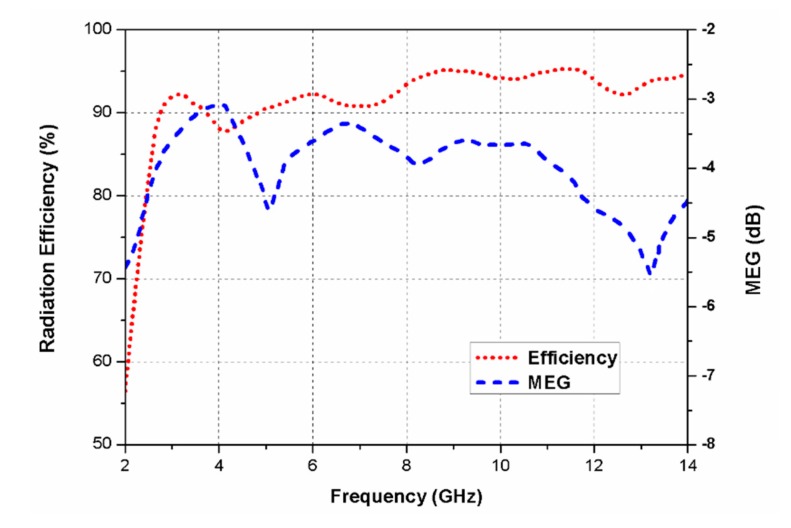
Radiation efficiency and mean effective gain (MEG) of the proposed antenna.

Figure 12 displays the simulated radiation efficiency of the proposed UWB MIMO antenna. The highest efficiency of 96% was obtained at 11.5 GHz. Furthermore, in Figure 11, the mean effective gain (MEG) of the antenna is presented, which was computed by comparing the mean power level of the test antenna with a reference antenna. It describes the impact caused by the antenna on the link budget and is used to evaluate the performance of an antenna in a real-time environment. MEG is the average received power, which defines first-order statistics of the signal envelope in the Rayleigh fading environment. It also shows how a deterministic device, like an antenna, will perform in a stochastic channel [39,40].

The apparent diversity gain (ADG) was calculated to study the rise in the quality of a signal, on introducing diversity in the multi-antenna systems [41]. ADG is a time-averaged quantity, which is used to measure the diversity performance of the antenna. It decreases with the increasing mean gain difference of the diversity branches and correlation coefficient. ADG can also be seen as the improvement in signal-to-noise ratio for a given outage time and is represented in decibels (dB). It can be calculated by using

(4) $$\mathrm{ADG}=10\sqrt{1-{\left|\rho_{e}\right|}^{2}}$$

For the proposed antenna, the performance at frequencies 4 GHz, 8 GHz, and 12 GHz was calculated and is represented in Table 2, where the ADG was observed to be greater than 9.9 dB. The isolation and ECC values are also shown in Table 2, at frequencies 4 GHz, 8 GHz, and 12 GHz.

**Figure 13:**
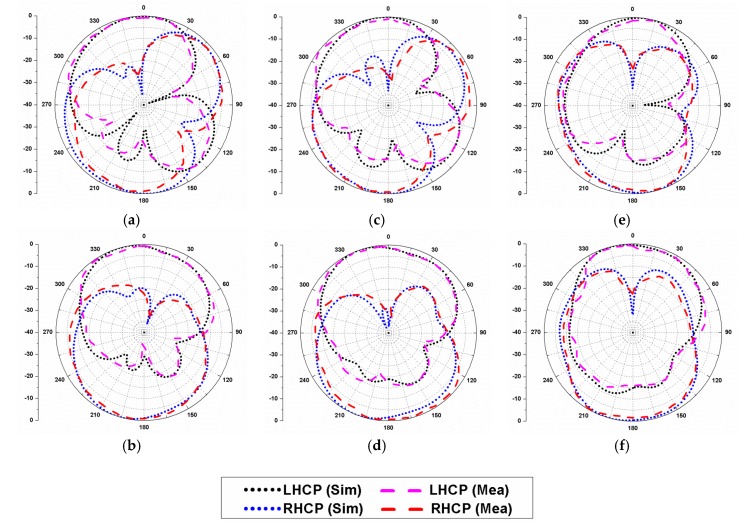
Radiation characteristics of the CP antenna: (**a**) 4.5 GHz, *φ* = 0°; (**b**) 4.5 GHz, *φ* = 90°; (**c**) 5 GHz, *φ* = 0°; (**d**) 5 GHz, *φ* = 90°; (**e**) 5.5 GHz, *φ* = 0°; (**f**) 5.5 GHz, *φ* = 90°.

The radiation patterns at 4.5 GHz, 5 GHz, and 5.5 GHz are illustrated in Figure 13, and the presented plots confirm the LHCP radiation behaviour of the presented antenna. The simulated and experimental results were marked in good concurrence with each other, and a minor difference was due to the cable effects, soldering, and fabrication error.

Table 3 compares the performance of the designed quad-port MIMO antenna with other reported MIMO/diversity antenna geometries [20,21,22,23,24,25,26,27,28,29,30,31]. Large-size dual-port or triple-port antennas with small impedance bandwidth are reported in [20,21,22,23,24,25,26,27,28]. In [22], the antenna possesses poor inter-element isolation, but good 3-dB ARBW. In [29,30], four-port antennas with smaller ARBW and impedance bandwidth are reported. The MIMO antennas with linear polarization characteristics are reported in [21,31]. The proposed MIMO geometry contained four antenna elements, packed within a small space with improved inter-element isolation, hence offering a robust diversity scheme with better link reliability, high throughput, and efficiency. This comparison shows that the proposed antenna was of small size and covered a broad range of axial ratio and impedance bandwidth. Also, the common ground plane and planar geometry help in the easy integration of the antenna on MMICs.

## 4. Conclusions

In this article, a small-size, low-profile, CP quad-port UWB MIMO/diversity antenna design with wide ARBW is presented. The antenna design consists of four identical microstrip line-fed antenna elements, and the circular polarization is achieved using the protruded hexagonal stubs. The compact size helps in easy integration of the antenna into wireless communication devices with limited space constraints. An ARBW of 2.7 GHz and impedance bandwidth of 11.3 GHz are realized with the proposed antenna design. The calculated ADG, ECC, and isolation lie within acceptable levels, which reflects the diversity performance of the presented antenna. Thus, the presented antenna overcomes the shortcomings of the MIMO antennas reported so far and could be useful for WLAN, WiMAX, S-, C-, and X-band applications.

## Figures and Tables

**Table 1:** Design parameters of the antenna element/multiple-input–multiple-output (MIMO) antenna.

Parameter	Dimension (mm)	Parameter	Dimension (mm)
L	20	L_1_	45
W	20	W_1_	45
l_1_	12.12	l_4_	1
l_2_	10	l_5_	1
l_3_	1.47	l_6_	1
d_1_	17.5	d_3_	1
d_2_	3	d_4_	1
s_1_	4	d_5_	1

**Table 2:** Performance features of the proposed CP UWB MIMO antenna.

Frequency (GHz)	Isolation (dB)	ADG (dB)	ECC
4	>19	9.98	<0.01
8	>26	10	<0.01
12	>26	9.9	<0.01

**Table 3:** Performance comparison between this work and previous works on the MIMO antenna.

Ref.	No. of Radiators	3-dB Axial Ratio Frequency (GHz)	ARBW (GHz)/(%)	Antenna Size (mm^3^)	−10 dB Impedance Frequency (GHz)	Effective Size	Impedance BW (GHz)/(%)	Gain (dB)	Isolation (dB)
[20]	2	2.4–2.5	0.1/4	30 × 30 × 4	2.38–2.52	0.24λ_0_ × 0.24λ_0_	0.14/6	—	>17
[21]	2	—	—	100 × 50 × 1.54	1.65–1.9, 2.68–6.25	0.55λ_0_ × 0.27λ_0_	0.25, 3.57/14, 80	6.78	>15
[22]	2	1.1–1.7	0.6/43	94 × 94 × 1.6	1.02–1.72	0.32λ_0_ × 0.32λ_0_	0.7/51	2.65	>14
[23]	2	5.5–5.9	0.4/7	30 × 25 × 1.524	5–6.8	0.5λ_0_ × 0.42λ_0_	1.8/31	4.4	>14
[24]	2	5.2–6.3	1.1/18	13.7 × 36.2 × 15	5.2–6.3	0.24λ_0_ × 0.64λ_0_	1.1/18	5.8	>20
[25]	2	2.04–2.57	0.53/23	66 × 66	1.82–2.57	0.4λ_0_ × 0.4λ_0_	0.75/34	4	>24
[26]	2	6.5, 7.5	—	58 × 29 × 1.6	3.1–10.6	0.6λ_0_ × 0.3λ_0_	7.5/109	5.8	>27
[27]	2	3.58–4.4	0.82/20	35 × 35 × 26.1	3.5–4.95	0.41λ_0_ × 0.41λ_0_	1.45/34	6.2	>28
[28]	3	5.52–5.64	0.12/2	29 × 48 × 1.6	5.5–6.25	0.53λ_0_ × 0.88λ_0_	0.75/13	4	>15
[29]	4	5.772–5.864	0.092/2	97 × 27.69 × 1.524	5.49–6.024	1.77λ_0_ × 0.51λ_0_	0.534/9	5.34	>33
[30]	4	3.56–3.67, 5.16–5.29	0.11, 0.13/3, 2	165 × 165 × 1.6	1.71–1.88, 3.3–3.7, 5.15–5.35	0.94λ_0_ × 0.94λ_0_	0.17, 0.4, 0.2/9, 11, 4	5	>45
[31]	4	—	—	75.19 × 75.19 × 1.6	3.1–17.3	0.78λ_0_ × 0.78λ_0_	10.2/139	5.5	>13
Prop.	4	3.8–6.5	2.7/52	45 × 45 × 1.6	2.2–13.5	0.33λ_0_ × 0.33λ_0_	11.3/144	6.8	>18
